# Improving the use of research evidence in guideline development: introduction

**DOI:** 10.1186/1478-4505-4-12

**Published:** 2006-11-20

**Authors:** Andrew D Oxman, Atle Fretheim, Holger J Schünemann, SURE

**Affiliations:** 1 Norwegian Knowledge Centre for the Health Services, P.O. Box 7004, St. Olavs plass, N-0130 Oslo, Norway; 2 INFORMA, S.C. Epidemiologia, Istitituto Regina Elena, Via Elio Chianesi 53, 00144 Rome, Italy; 3 Subcommittee on the Use of Research Evidence (SURE) of the WHO Advisory Committee on Health Research (ACHR)

## Abstract

In 2005 the World Health Organisation (WHO) asked its Advisory Committee on Health Research (ACHR) for advice on ways in which WHO can improve the use of research evidence in the development of recommendations, including guidelines and policies. The ACHR established the Subcommittee on the Use of Research Evidence (SURE) to collect background documentation and consult widely among WHO staff, international experts and end users of WHO recommendations to inform its advice to WHO. We have prepared a series of reviews of methods that are used in the development of guidelines as part of this background documentation. We describe here the background and methods of these reviews, which are being published in *Health Research Policy and Systems *together with this introduction.

## Background

In May of 2005 the 58^th ^World Health Assembly passed a resolution requesting the Director-General *"to undertake an assessment of WHO's internal resources, expertise and activities in the area of health research, with a view to developing a position paper on WHO's role and responsibilities in the area of health research, and to report through the Executive Board to the next World Health Assembly." *Related to these resolutions, WHO has asked the Advisory Committee on Health Research (ACHR) for advice on ways in which WHO can improve the use of research evidence in the development of recommendations, guidelines and policies.

The ACHR established a subcommittee to collect background documentation and consult widely among WHO staff, international experts and end users of WHO recommendations to inform this advice. The advice will focus on processes to ensure that WHO's recommendations are well informed by the best available research evidence. These processes range from how WHO sets priorities for the development of recommendations to how its recommendations are disseminated and implemented, including recommendations developed at WHO headquarters in Geneva, at its regional offices and in countries.

WHO from its inception has focused on research, which is mandated in its constitution, and has been a leading player in the global effort to strengthen ties between research and health development.

Given WHO's position as the world's leading public health agency, it is essential that the organisation, its leaders and its governing body ensure that its recommendations and actions are as well informed as possible by the best available research evidence. WHO has strived to do this for over 50 years with much success.

However, major new developments have occurred since WHO was established that have led governments around the world to reconsider the methods that they use to ensure that decisions about health care and public health are well informed by research evidence. This reflection and subsequent changes in how recommendations about health are developed have been driven by recognition of gaps between available research evidence and what is done in practice, variations in practice and outcomes, concerns about the quality of health care, and rising health care costs.

Increasingly governments, professional and consumer organisations are demanding more rigorous processes to ensure that health decisions are well informed by the best available research evidence. The processes, in contrast with traditional approaches that rely heavily on the opinions of experts, demand systematic and transparent approaches to access, synthesise and interpret research evidence; and to integrate that evidence with the other information, values and judgements to formulate recommendations. The need for more rigorous processes is underscored by evidence of inconsistencies between the available evidence and expert recommendations [1,2], insufficient use of the available evidence [3,4], and other insufficiencies in how guidelines and recommendations are developed [5-12]. Similar criticisms have been raised and calls have been made for better use of research evidence for health care management and policy making, as well [13-15].

WHO has the opportunity and the mandate to capitalise on these advances and to assist its member states to do so. This is essential to ensure that decisions about health are well informed by research evidence, and that these decisions lead to effective, efficient and equitable actions towards achieving WHO's goal: the attainment by all peoples of the highest possible level of health.

As part of the background documentation to inform ACHR's advice to WHO we have prepared a series of reviews on the following topics:

• Guidelines for guidelines [16]

• Priority setting [17]

• Group composition and consultation process [18]

• Managing conflicts of interest [19]

• Group processes [20]

• Determining which outcomes are important [21]

• Deciding what evidence to include [22]

• Synthesis and presentation of evidence [23]

• Grading evidence and recommendations [24]

• Integrating values and consumer involvement [25]

• Incorporating considerations of cost-effectiveness, affordability and resource implications [26]

• Incorporating considerations of equity [27]

• Adaptation, applicability and transferability [28]

• Reporting guidelines [29]

• Disseminating and implementing guidelines [30]

• Evaluation [31]

We have used the term 'guidelines' broadly to include a wide range of recommendations that WHO makes, including clinical, public health and health policy recommendations. Although much of the literature that we have reviewed has focused on clinical practice guidelines, we have tried to incorporate corresponding literature for public health guidelines and health policy recommendations.

The reviews are not full systematic reviews, although we have aimed to be reasonably systematic and transparent about the methods we have used and the basis for the recommendations that we have made. For each review we began with a series of key questions that were vetted amongst the authors and the ACHR Subcommittee on the Use of Research Evidence (SURE). The first author of each review conducted searches for relevant literature and prepared the first draft. The search strategies that were used are summarised in each review. We did not always conduct exhaustive reviews. We tried first to identify existing systematic reviews that addressed the questions that we asked and, secondarily, if we did not find a systematic review, relevant methodological research. When there was a paucity of research, we have also included some descriptive literature or, in some cases, evidence that was not directly related to guidelines development.

Each review includes short summaries of what WHO and other organisations are doing, our key findings in relationship to each of the questions that we asked, a discussion of those findings and some suggestions for further work that is needed. Our answers to the key questions that we asked are summarised in the abstract of each review.

A draft of each review was first discussed and revised by the authors. The reviews were also circulated to the ACHR SURE members and discussed by the subcommittee. After peer review the articles were revised by the authors and updated if necessary. We are grateful to the editors of *Health Research Policy and Systems *for agreeing to publish these papers in their journal. In addition to benefiting from their editorial support, this has enabled us to take advantage of the BioMed Central's open peer review system to help ensure the quality of our reviews and advice. We also believe that these reviews are of wide interest to other organisations and individuals that are responsible for developing guidelines or health policy.

In addition to this series of reviews that is being published in *Health Research Policy and Systems*, we have conducted reviews of what WHO is currently doing, using both document analyses and interviews and we have conducted a survey of initiatives around the world that support the use of research evidence in developing guidelines or health policy. We have referred to these reports, which are being published separately, where relevant in the reviews in this series.

Preliminary advice from the ACHR has already been discussed with the leadership of WHO. It has been positively received and, to some extent, is being acted upon already. Before delivering our final report and advice to WHO, we will consult with a reference panel and others within and outside of WHO. We look forward to working with WHO to help implement this advice. We hope that it will assist WHO to better serve its member states by ensuring that its recommendations are well-informed by the best available research evidence, and by enabling those responsible for making decisions to make well-informed choices.

## Competing interests

ADO and AF work for the Norwegian Knowledge Centre forthe Health Services, an agency funded by the Norwegian government that produces systematic reviews and health technology assessments. All three authors are contributors to the Cochrane Collaboration. ADO and HJS are members of the GRADE Working Group. HJS is documents editor and chair of the documents development and implementation committee for the American Thoracic Society and senior editor of the American College of Chest Physicians' Antithrombotic and Thrombolytic Therapy Guidelines.

## Authors' contributions

ADO prepared the first draft of this introduction. AF and HJS contributed to drafting and revising it.

## References

[B1] Oxman AD, Guyatt GH (1993). The science of reviewing research. Annals of the New York Academy of Science.

[B2] Antman EM, Lau J, Kupelnick B, Mosteller F, Chalmers TC (1992). A comparison of results of meta-analyses of randomized control trials and recommendations of clinical experts. Treatments for myocardial infarction. JAMA.

[B3] Silagy CA, Stead LF, Lancaster T (2001). Use of systematic reviews in clinical practice guidelines: case study of smoking cessation. BMJ.

[B4] Vigna-Taglianti F, Vineis P, Liberati A, Faggiano F (2006). Quality of systematic reviews used in guidelines for oncology practice. Annals of Oncology.

[B5] Bradbury J (1999). Storm over WHO-ISH hypertension guidelines. Lancet.

[B6] Horton R (2002). WHO: the casualties and compromises of renewal. Lancet.

[B7] Laing R, Waning B, Gray A, Ford N, t Hoen E (2003). 25 years of the WHO essential medicines lists: progress and challenges. Lancet.

[B8] McCarthy M (2005). Critics slam draft WHO report on homoeopathy. Lancet.

[B9] Oxman AD, Lavis JN, Fretheim A The use of evidence in WHO recommendations. Lancet.

[B10] Shaneyfelt TM, Mayo-Smith MF, Rothwangl J (1999). Are guidelines following guidelines? The methodological quality of clinical practice guidelines in the peer-reviewed medical literature. JAMA.

[B11] Grilli R, Magrini N, Penna A, Mura G, Liberati A (2000). Practice guidelines developed by specialty societies: the need for a critical appraisal. Lancet.

[B12] Grol R, Dalhuijsen J, Thomas S, Veld C, Rutten G, Mokkink H (1998). Attributes of clinical guidelines that influence use of guidelines in general practice: observational study. BMJ.

[B13] Lavis JN, Posada FB, Haines A, Osei E (2004). Use of research to inform policymaking. Lancet.

[B14] Lavis JN, Davies H, Oxman AD, Denis JL, Golden-Biddle K, Ferlie E (2005). Towards systematic reviews that inform health care management and policy-making. J Health Serv Res Policy.

[B15] Sheldon TA (2005). Making evidence synthesis more useful for management and policy-making. J Health Serv Res Policy.

[B16] Schünemann HJ, Fretheim A, Oxman AD (2006). Improving the Use of Research Evidence in Guideline Development: 1. Guidelines for guidelines. Health Res Policy Syst.

[B17] Oxman AD, Schünemann HJ, Fretheim A (2006). Improving the Use of Research Evidence in Guideline Development: 2. Priority setting. Health Res Policy Syst.

[B18] Fretheim A, Schünemann HJ, Oxman AD (2006). Improving the Use of Research Evidence in Guideline Development: 3. Group composition. Health Res Policy Syst.

[B19] Boyd E, Bero L (2006). Improving the Use of Research Evidence in Guideline Development: 4. Managing conflicts of interest. Health Res Policy Syst.

[B20] Fretheim A, Schünemann HJ, Oxman AD (2006). Improving the Use of Research Evidence in Guideline Development: 5. Group processes. Health Res Policy Syst.

[B21] Schünemann HJ, Oxman AD, Fretheim A (2006). Improving the Use of Research Evidence in Guideline Development: 6. Determining which outcomes are important. Health Res Policy Syst.

[B22] Oxman AD, Fretheim A, Schünemann HJ (2006). Improving the Use of Research Evidence in Guideline Development: 7. Deciding what evidence to include. Health Res Policy Syst.

[B23] Oxman AD, Schünemann HJ, Fretheim A (2006). Improving the Use of Research Evidence in Guideline Development: 8. Synthesis and presentation of evidence. Health Res Policy Syst.

[B24] Schünemann HJ, Fretheim A, Oxman AD (2006). Improving the Use of Research Evidence in Guideline Development: 9. Grading evidence and recommendations. Health Res Policy Syst.

[B25] Schünemann HJ, Fretheim A, Oxman AD (2006). Improving the Use of Research Evidence in Guideline Development: 10. Integrating values and consumer involvement. Health Res Policy Syst.

[B26] Edejer TTT (2006). Improving the Use of Research Evidence in Guideline Development: 11. Incorporating considerations of cost-effectiveness, affordability and resource implications. Health Res Policy Syst.

[B27] Oxman AD, Schünemann HJ, Fretheim A (2006). Improving the Use of Research Evidence in Guideline Development: 12. Incorporating considerations of equity. Health Res Policy Syst.

[B28] Schünemann HJ, Fretheim A, Oxman AD (2006). Improving the Use of Research Evidence in Guideline Development: 13. Adaptation, applicability and transferability. Health Res Policy Syst.

[B29] Oxman AD, Fretheim A, Schünemann HJ (2006). Improving the Use of Research Evidence in Guideline Development: 14. Reporting guidelines. Health Res Policy Syst.

[B30] Fretheim A, Schünemann HJ, Oxman AD (2006). Improving the Use of Research Evidence in Guideline Development: 15. Disseminating and implementing guidelines. Health Res Policy Syst.

[B31] Oxman AD, Fretheim A, Schünemann HJ (2006). Improving the Use of Research Evidence in Guideline Development: 16. Evaluation. Health Res Policy Syst.

